# Mouse *Survival Motor Neuron* Alleles That Mimic *SMN2* Splicing and Are Inducible Rescue Embryonic Lethality Early in Development but Not Late

**DOI:** 10.1371/journal.pone.0015887

**Published:** 2010-12-29

**Authors:** Suzan M. Hammond, Rocky G. Gogliotti, Vamshi Rao, Ariane Beauvais, Rashmi Kothary, Christine J. DiDonato

**Affiliations:** 1 Human Molecular Genetics Program, Children's Memorial Research Center, Chicago, Illinois, United States of America; 2 Department of Pediatrics, Feinberg School of Medicine, Northwestern University, Chicago, Illinois, United States of America; 3 Ottawa Hospital Research Institute, Ottawa, Canada; 4 The University of Ottawa Center for Neuromuscular Disease, Ottawa, Canada; 5 Department of Cellular and Molecular Medicine, University of Ottawa, Ottawa, Canada; 6 Department of Medicine, University of Ottawa, Ottawa, Canada; Columbia University, United States of America

## Abstract

Spinal muscular atrophy (SMA) is caused by low survival motor neuron (SMN) levels and patients represent a clinical spectrum due primarily to varying copies of the *survival motor neuron-2* (*SMN2*) gene. Patient and animals studies show that disease severity is abrogated as SMN levels increase. Since therapies currently being pursued target the induction of SMN, it will be important to understand the dosage, timing and cellular requirements of SMN for disease etiology and potential therapeutic intervention. This requires new mouse models that can induce SMN temporally and/or spatially. Here we describe the generation of two hypomorphic *Smn* alleles, *Smn^C-T-Neo^* and *Smn^2B-Neo^*. These alleles mimic *SMN2* exon 7 splicing, titre Smn levels and are inducible. They were specifically designed so that up to three independent lines of mice could be generated, herein we describe two. In a homozygous state each allele results in embryonic lethality. Analysis of these mutants indicates that greater than 5% of Smn protein is required for normal development. The severe hypomorphic nature of these alleles is caused by inclusion of a *loxP*-flanked *neomycin* gene selection cassette in *Smn* intron 7, which can be removed with Cre recombinase. *In vitro* and *in vivo* experiments demonstrate these as inducible *Smn* alleles. When combined with an inducible *Cre* mouse, embryonic lethality caused by low Smn levels can be rescued early in gestation but not late. This provides direct genetic evidence that a therapeutic window for SMN inductive therapies may exist. Importantly, these lines fill a void for inducible *Smn* alleles. They also provide a base from which to generate a large repertoire of SMA models of varying disease severities when combined with other *Smn* alleles or *SMN2*-containing mice.

## Introduction

The *survival motor neuron* (*SMN*) gene is ubiquitously expressed and encodes an essential protein that is required by all cells [1]. Low levels of SMN cause proximal spinal muscular atrophy (SMA), an autosomal recessive disease, and a common genetic cause of infant mortality [2], [3]. It is pathologically characterized by selective loss of lower motor neurons within the spinal cord, causing progressive muscle atrophy due to denervation. Proximal muscles within the limbs and trunk are more affected than distal muscle groups, but ultimately all muscles succumb to denervation causing paralysis, respiratory deficiency and ultimately death.

Clinically, SMA is heterogeneous and has been divided into three major groups based upon age at onset and achieved motor milestones [4], [5]. Genetically SMA is homogenous in that all forms of the disease are caused by homozygous deletion, rare subtle mutations, or gene conversion of the *survival motor neuron-1* (*SMN1*) gene with concurrent retention of a linked paralog, *survival motor neuron-2* (*SMN2*) [2], [6], [7]. Both *SMN* genes reside in a duplicated genomic region at 5q13, are transcribed, translated and 99.9% identical [2], [8], [9]. The key difference is a single, translationally silent nucleotide transition (C to T) at the +6 position within exon 7 that functionally distinguishes *SMN1* from *SMN2* and prevents *SMN2* from fully compensating for *SMN1* loss [2], [9], [10]. *SMN1* contains a “C” nucleotide and produces full-length SMN transcripts (*FL-SMN*). In contrast, *SMN2* contains a “T” nucleotide and primarily produces transcripts that lack exon 7 (*SMNΔ7*) and a small amount of *FL-Smn*. This is due to the simultaneous disruption of an ASF/SF2 exon splice enhancer (ESE) and creation of an exon splice silencer (ESS) in *SMN2* [11], [12].

The *SMN2* copy number in an individual can vary from one to six and it is this variability that is mainly responsible for the clinical spectrum seen in SMA patients [13]. Since every SMA patient has at least one functioning *SMN2* gene, it has become a target for therapeutic interventions, and most pre-clinical studies have focused on up-regulating SMN levels by some means [14], [15], [16], [17], [18], [19], [20], [21], [22], [23], [24]. An important point of all SMN-dependent therapies is an understanding of when, where and how much SMN induction is required, and how this might change for the various clinical forms of SMA. The dosage, timing and cellular requirements of SMN in different tissues should not be overlooked as there is mounting evidence in humans and mice that suggest non-motor neuron targets such as heart, autonomic and vascular systems may require consideration [25], [26], [27], [28]. Although some data is already available and demonstrates a therapeutic window of opportunity to affect a benefit for severe SMA mice [15], [17], [29], a new panel of mice is required in which SMN can be induced temporally and/or spatially to refine and extend current results.

In this study, we report the generation and characterization of two *Smn* progenitor alleles, *Smn^C-T-Neo^* and *Smn^2B-Neo^*. They were designed to stimulate *Smn* exon 7 alternative splicing, which normally does not occur in the mouse [30], [31]. *Smn^C-T-Neo^* and *Smn^2B-Neo^* are severe hypomorphs that cause embryonic lethality when in a homozygous state due to the presence of a *loxP*- flanked *neomycin* (*neo*) gene resistance cassette that hinders *Smn* expression. However, in the presence of Cre recombinase, the embryonic lethality can be rescued by *neo* excision, while still maintaining *Smn* exon 7 alternative splicing via our introduced mutations. *In vitro* and *in vivo* experiments demonstrate the utility of these mice to be used as inducible *Smn* alleles when combined with *Cre* transgenic lines. Using a tamoxifen-inducible *Cre* line we show that embryonic lethality can be rescued early in gestation but not late. As a final point, the *Smn^C-T-Neo^* and *Smn^2B-Neo^* lines were specifically designed to be progenitor alleles, so that potentially three useful lines of mice could be generated from each targeting event. Importantly, these lines alter the endogenous *Smn* locus so they mimic *SMN2* exon 7 alternative splicing and the situation of SMA patients, which is reduction of Smn protein levels, not absence of protein. When used as inducible *Smn* alleles, they increase Smn levels under the normal regulation of the endogenous locus, while still mimicking *SMN2* splicing.

## Results

### Generation and germline transmission of *Smn^C-T-Neo^* and *Smn^2B-Neo^* alleles

**Figure 1:**
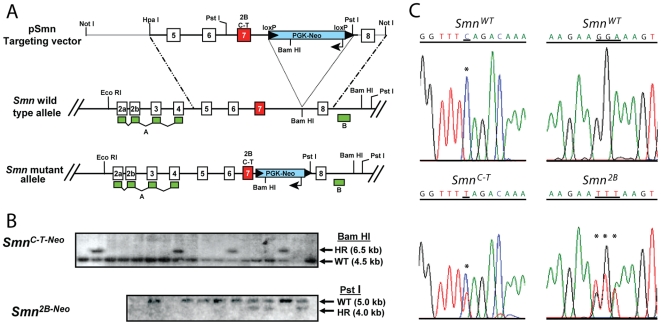
Generation of mutant *Smn* alleles. (**A**) Gene targeting strategy to introduce the C-T and 2B mutation into *Smn* exon 7 using the gene targeting vectors pSmnC-T-Neo and pSmn2B-Neo. (**B**) Southern blot analysis of BamH I and Pst I digested DNA from *neo* resistant ES cell clones identified homologous recombinants. Two clones from each were used to perform blastocyst injections. (**C**) Germline transmission of *Smn^C-T-Neo^* and *Smn^2B-Neo^* alleles were determined by direct sequencing of *Smn* exon 7 PCR products from heterozygous mice. The C-T mutation corresponds to the nucleotide transition within exon 7 of the *SMN2* gene. The 2B mutation corresponds to a mutation within the splice enhancer region 2B, changing GGA to TTT [32].

Based on our previous studies we designed two replacement vectors, p(Smn^C-T-Neo^) and p(Smn^2B-Neo^) and introduced two different mutations into the endogenous *Smn* locus by homologous recombination. The first mimics human *SMN2* and is a C-T transition at position 6 of exon 7, referred to hereafter as the C-T mutation. The second mutation alters the central portion of the ESE where hTra2-Beta1 binds *Smn* exon 7 (GGA to TTT), and we refer to this mutation as the 2B mutation [32] (Figure 1A). It is known that this binding site is important for *SMN* exon 7 processing [33]. For both replacement vectors the positive selection cassette, *pgk-neo*, was inserted into the unique BamHI site ∼180 bp distal to exon 7 in the antisense orientation to *Smn* transcription. This was specifically done as an additional way to potentially hinder *Smn* processing. We also flanked (“floxed”) the *pgk-neo* cassette with *loxP* sites so that it could be excised with *Cre* recombinase in future experiments to leave the endogenous locus with a minimal amount of alteration.

Homologous recombinant embryonic stem cell clones were identified by Southern blot hybridization (Figure 1B) and two independent clones for each construct used to generate chimeras. Germline transmission was confirmed through direct sequencing of *Smn* exon 7 PCR products (Figure 1C). We refer to these progenitor lines as *Smn^C-T-Neo^* (official name *Smn1^tm2Cdid^*) and *Smn^2B-Neo^* (official name *Smn1^tm1Cdid^*) as they retain the floxed *pgk-neo* selection cassette within *Smn* intron 7. All subsequent experiments reported herein were performed after backcrossing to C57Bl/6 mice for at least three generations.

### *Smn^C-T-Neo^* and *Smn^2B-Neo^* alleles express very small amounts of Smn

**Figure 2:**
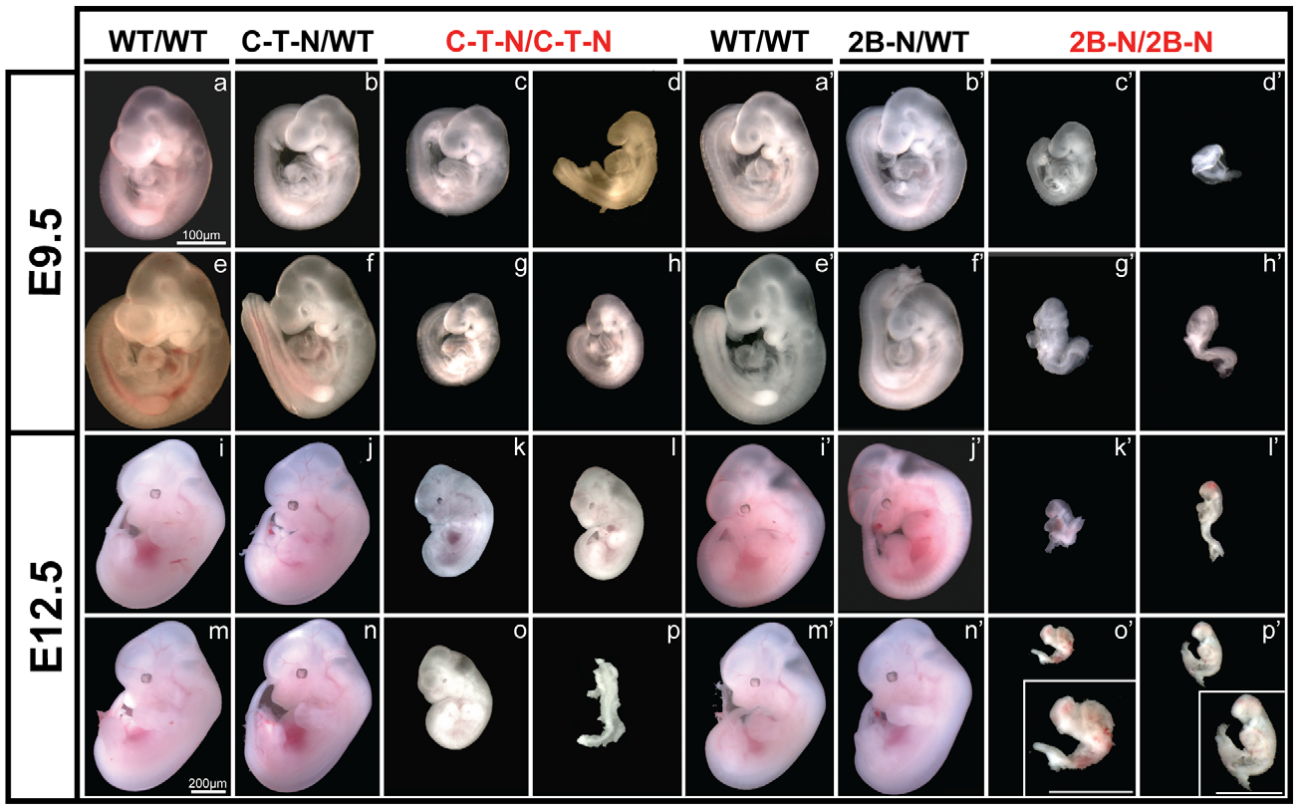
Whole mount analysis of embryos. *Smn^2B-Neo^* or *Smn^C-T-Neo^* heterozygotes were intercrossed and embryos obtained at either E9.5 or E12.5 for whole-mount analysis and genotyping. (***a–h***) E9.5 *Smn^C-T-Neo^* embryos. Heterozygotes (C-T-N/WT) are identical to wild type (WT/WT) littermates. Homozygotes (C-T-N/C-T-N) are small but alive and larger than the *Smn^2B-Neo/2B-Neo^* (2B-N/2B-N) homozygotes. (***i–p***) E12.5 *Smn^C-T-Neo^* embryos. Homozygotes are extremely small compared to controls and many are being reabsorbed as shown in panel (p). (**a'–h'**) E9.5 *Smn^2B-Neo^* embryos. Heterozygotes (2B-N/WT) are identical to wild type (WT/WT) littermates. Homozygotes (2B-N/2B-N) are developmentally retarded though still alive with signs of lethality clearly present before this period in some embryos that did not allow for genotyping (Table 1). (***i'–p'***) E12.5 *Smn^2B-Neo^* embryos. All homozygous mutant embryos are undergoing resorption. Insets in o' and p' are magnified images of embryos in panel. Scale for all E9.5 embryos is 100 µM and for E12.5 200 µM.

To evaluate the effects of C-T-Neo and 2B-Neo mutations on *Smn* exon 7 processing, all major organs including spinal cord and skeletal muscle from postnatal tissues of heterozygous mice were analyzed by reverse transcription-polymerase chain reaction (RT-PCR). *Smn^C-T-Neo/WT^* mice produced both *FL-Smn* and *Δ7Smn* transcripts as did *Smn^2B-Neo/WT^* mice (data not shown). We performed a series of intercrosses for each allele during the course of this study. Of 229 and 281 pups genotyped at weaning from *Smn^C-T-Neo/WT^* and *Smn^2B-Neo/WT^* intercrosses, respectively, none were found to be homozygous (Table 1). To identify when during development *Smn^C-T-Neo/C-T-Neo^* and *Smn^2B-Neo/2B-Neo^* embryos were dying, we performed a series of timed matings. For *Smn^C-T-Neo/WT^* intercrosses at 9.5 days post-coitum (E9.5), a total of 58 embryos were analyzed. While near mendelian ratios of wild type (*Smn^WT/WT^*), heterozygous (*Smn^C-T-Neo/WT^*) and homozygous mutants (*Smn^C-T-Neo/C-T-Neo^*) were identified by genotyping, developmental delays of homozygous mutant *Smn^C-T-Neo/C-T-Neo^* embryos (11/58; 19%) were visible (Table 1 and Figure 2, panels a–h). Although at E12.5 we could still detect *Smn^C-T-Neo/C-T-Neo^* embryos (5/56; 9%), it was clear that all were developmentally delayed and some were undergoing resorption (Table 1 and Figure 2, panels i–p). By E15.5 only wild type and heterozygous *Smn^C-T-Neo/WT^* embryos were present (Table 1).

**Table 1:** Genotype of *Smn^C-T-Neo^* and *Smn^2B-Neo^* intercross progeny.

Age	# of Litters	Total Pups	+/+ (%)	+/− (%)	−/− (%)	unknown identity (%)	χ
***Smn^C-T-Neo^*** ** intercross progeny**							
**1 month**	39	229	80 (35)	144 (63)	0 (0)	5 (2)	83.6 (P<0.001)
**E15.5**	5	33	15 (45)	18 (54.5)	0 (0)	0 (0)	13.9 (P<0.001)
**E12.5**	8	56	14 (25)	28 (50)	5 (9)	9 (16)	5.17 (P>0.05)
**E9.5**	6	58	16 (27.5)	22 (38)	11 (19)	9 (15.5)	1.5 (P<0.05)
***Smn^2B-Neo^*** ** intercross progeny**							
**1 month**	44	281	109 (39)	170 (60.5)	0 (0)	2 (0.7)	98.5 (P<0.001)
**E15.5**	6	44	22 (50)	22 (50)	0 (0)	0 (0)	22 (P<0.001)
**E12.5**	7	63	5 (8)	36 (57)	9 (14)	13 (21)	11.6 (P<0.01)
**E9.5**	9	84	21 (25)	38 (45)	14 (17)	11 (13)	1.46 (P>0.05)

(+) denotes wild type, (−) denotes either C-T-Neo or 2B-Neo.

Our results for embryo analysis from *Smn^2B-Neo/WT^* intercrosses were more dramatic. We analyzed 84 embryos at E9.5 and while we could detect *Smn^2B-Neo/2B-Neo^* homozygotes (14/84; 17%), these embryos were more developmentally delayed than the *Smn^C-T-Neo/C-T-Neo^* embryos and were starting to be reabsorbed (Table 1 and Figure 2 panels a'–h'). At E12.5 all *Smn^2B-Neo/2B-Neo^* homozygotes that we could detect were dead and at different levels of resorption (Table 1 and Figure 2, panels i'–p'). By E15.5 only wild type and heterozygous *Smn^2B-Neo/WT^* embryos were present (Table 1).

**Figure 3:**
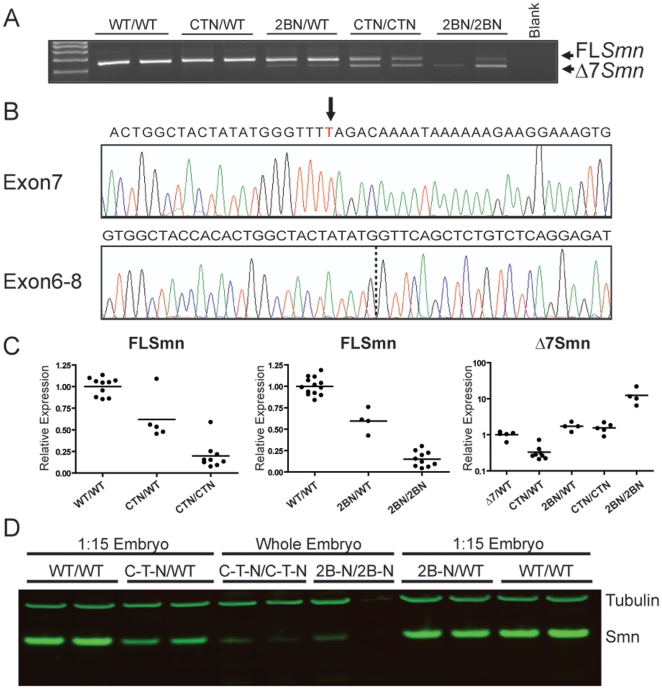
*Smn* transcript analysis analysis of E9.5 embryos. (**A**) RT-PCR of E9.5 embryos comparing wild type, heterozygotes, and homozygotes. Both *FL-Smn* and *Δ7Smn* transcripts are amplified from cDNA of mice that are heterozygous and homozygous for the mutant alleles. (**B**) Direct sequencing of *FL-Smn* and *Δ7Smn* RT-PCR products derived from *Smn^C-T-Neo/C-T-Neo^* mutants. The “T” denoted with an arrow above it, represents the C-T mutation. Dotted line within exon6-8 sequence represents the junction between exon 6 and exon 8. (**C**) qRT-PCR results of E9.5 embryo for *FL-Smn* and *Δ7Smn* transcripts. *FL-Smn* transcripts for C-T-Neo and 2B-Neo were compared to wild type and heterozygous embryos within their own intercrosses and litters to control for variability. Spinal cord (S.C.) cDNA from a 6-month Δ7/WT mouse was used to compare *Δ7Smn* transcripts from C-T-Neo and 2B-Neo heterozygous and homozygous embryos. Wild type mice do not express *Δ7Smn* and are not shown on the graph. Each data point on the graphs represent individual embryos and depiction of variability between embryos. (**D**) Immunoblot analysis of Smn expression from individual E9.5 embryos derived from *Smn^C-T-Neo^* or *Smn^2B-Neo^* intercrosses. 15 times less protein was used for the controls to avoid overloading SMN while simultaneously detecting it in the mutants. Note the variation in Smn levels from individual mutant embryos. Lower Smn levels correlated with more severe phenotypes (see Figure 2). To achieve this sensitivity, Smn detection was performed on a Li-COR Odyssey infrared imaging system. Abbreviations: (WT) *Smn^WT/WT^*, (C-T-N/WT) *Smn^C-T-Neo/WT^*, (2B-N/WT) *Smn^2B-Neo/WT^*, (C-T-N/C-T-N) *Smn^C-T-Neo/C-T-Neo^*, (2B-N/2B-N) *Smn^2B-Neo/2B-Neo^*, (Δ7/WT) *Smn^Δ7/WT^*.

The embryonic lethality of *Smn^C-T-Neo/C-T-Neo^* and *Smn^2B-Neo/2B-Neo^* embryos occurs later than *Smn^Δ7/Δ7^* embryos, who die at E7.5 [34], [35]. This suggested that a small amount of *FL-Smn* was being produced. To determine whether this was the case, we analyzed RNA from E9.5 wild type, heterozygous and homozygous *Smn^C-T-Neo^* and *Smn^2B-Neo^* embryos by RT-PCR. Both *FL-Smn* and *Δ7Smn* were produced from heterozygous and homozygous *Smn^C-T-Neo^* and *Smn^2B-Neo^* embryos in contrast to wild type *Smn* embryos (*Smn^WT^*) (Figure 3A). Furthermore, the *Smn^C-T-Neo^* allele consistently produced more transcripts that contained *Smn* exon 7 than the *Smn^2B-Neo^* allele. Direct sequencing of the *FL-Smn* amplicon from *Smn^C-T-Neo/C-T-Neo^* embryos identified only the mutant “T” nucleotide at position +6 of *Smn* exon 7, as would be expected from homozygous mutant embryos (Figure 3B). Likewise, *FL-Smn* amplicons from *Smn^2B-Neo/2B-Neo^* embryos only expressed the 2B (TTT) mutation (data not shown).

To quantify the amount of *FL-Smn* and *Δ7Smn* transcripts that were produced in each of the genotypes from our intercross experiments, we designed two novel taqman assays for use in quantitative reverse transcription PCR (qRT-PCR). The first assay specifically detected *Smn* exon 7 in the presence of either the wild type, C-T or 2B mutation with similar efficiency. The second assay detected *Δ7Smn* transcripts. The values of *FL-Smn* from our *Smn^C-T-Neo^* and *Smn^2B-Neo^* genotypes were compared to the expression of *Smn^WT^* E9.5 embryos derived from the same intercross to reduce variability. Overall, the amount of *FL-Smn* varied with our different genotypes (Figure 3C and Table 2). *Smn^C-T-Neo/C-T-Neo^* embryos produced 20±5% *FL-Smn*, whereas *Smn^2B-Neo/2B-Neo^* embryos produced 15±3% and these values were not statistically significant from each other (p = 0.43). This indicated that each *Smn^C-T-Neo^* and the *Smn^2B-Neo^* allele produced ∼10% and ∼7.5% *FL-Smn* transcripts, respectively. These results are consistent with *Smn^C-T-Neo/WT^* (62±12%) and *Smn^2B-Neo/WT^* (59±7%) embryos, if you consider that ∼50% of *FL-Smn* transcripts are derived from the *Smn^WT^* allele (Figure 3C and Table 2).

**Table 2:** qRT-PCR analysis of E9.5 *Smn* embryos.

E9.5 embryos	*Fl-Smn* expression	*Δ7 Smn* expression
WT/WT	1.00±0.02	
C-T-Neo/WT	0.62±0.12	0.33±0.06
2B-Neo/WT	0.59±0.07	1.72±0.23
C-T-Neo/C-T-Neo	0.20±0.05	1.56±0.22
2B-Neo/2B-Neo	0.15±0.03	12.44±3.28
Δ7/WT (6 month S.C.)		1.00±0.10

S.C., spinal cord.

We also quantified the amount of *Δ7Smn* from our *Smn^C-T-Neo^* and *Smn^2B-Neo^* genotypes by comparing them to *Δ7Smn* transcripts derived from spinal cord samples of *Smn^Δ7/WT^* mice [34]. *Smn^2B-Neo/2B-Neo^*embryos expressed the greatest amount of *Δ7Smn* transcripts, whereas the *Smn^C-T-Neo/WT^* embryos expressed the least (Figure 3A and 3C). Using this data in conjunction with our *FL-Smn* data we were able to generate a *FL-Smn: Δ7Smn* ratio for each genotype using the ΔΔCt values. The ratio for *Smn^Δ7/WT^* mice was approximately equal to 1.0 (1.022±0.018) and served as a control. The wild type allele (WT) only produces transcripts that contain *Smn* exon 7, whereas the *Smn^Δ7^* allele only produces transcripts that lack exon 7 due to the absence of the exon in the genome of this allele [34]. In comparison, *Smn^C-T-Neo/WT^* mice produced about 12-fold more (11.98±0.929) *FL-Smn* than *Δ7Smn* transcripts. However, in homozygous embryos (*Smn^C-T-Neo/C-T-Neo^*) the ratio was almost equal to 1.0 (0.989±0.068) and this is consistent with the visual inspection of end-point RT-PCR (Figure 3A). Interestingly, we found the *FL-Smn: Δ7Smn* ratio to be 10-fold less in *Smn^2B-Neo/2B-Neo^* embryos (0.178±0.022) as compared to *Smn^C-T-Neo/C-T-Neo^* embryos even though the amount of *FL-Smn* produced from either the *Smn^C-T-Neo^* or *Smn^2B-Neo^* allele was not significantly different (p = 0.43). The change in ratio is due to the high levels of *Δ7Smn* transcripts that *Smn^2B-Neo/2B-Neo^* embryos produced and is consistent with the splicing pattern and transcript ratios of *SMN2* where the majority of *SMN2* transcripts lack exon 7.

To correlate *FL-Smn* transcripts to Smn protein we performed western blot analysis of single E9.5 embryos for each possible genotype. We had difficulty detecting Smn in homozygous mutant embryos, and the level varied between mutants of the same genotype (Figure 3D). In general, the level of Smn correlated with the severity of the mutant embryo when we retrospectively compared Smn levels to our whole mount mutant embryo images (Figure 3D and Figure 2). The level of Smn protein in *Smn^C-T-Neo/C-T-Neo^* embryos ranged from 2–5% and in *Smn^2B-Neo/2B-Neo^* embryos it was about 1–3%. This was an unexpected finding based on our *FL-Smn* expression data from *Smn^C-T-Neo/C-T-Neo^* and *Smn^2B-Neo/2B-Neo^* embryos and was most likely caused by the compromised physiological state of the SMA embryos, a lack of stability of higher order Smn complexes from low Smn levels and transcriptional and/or translational hindrance from the floxed *pgk-neo* cassette. Since the later was the only one that we could directly test, we removed the floxed *pgk-neo* cassette from the germline using homozygous *EIIa-Cre* transgenic mice that ubiquitously express Cre recombinase very early in embryogenesis [36]. This increased Smn protein levels from <5% for homozygous *Smn^C-T-Neo^* and *Smn^2B-Neo^* embryos to >30% for a single *Smn^C-T^* allele and ∼16% for a single *Smn^2B^* allele (data not shown and will be reported elsewhere). Collectively, these results confirm that our *Smn^C-T-Neo^* and *Smn^2B-Neo^* alleles are severe hypomorphs. They express very low amounts of Smn due to the nature of our introduced mutations as well as the presence of a floxed *pgk-neo* selection cassette that hinders *Smn* expression. These alleles are “repairable” and have the potential to be used as inducible *Smn* alleles that mimic *SMN2* splicing. To determine this directly, the following *in vitro* and *in vivo* experiments were designed to address the ability of *Smn^C-T-Neo^* and *Smn^2B-Neo^* alleles to be utilized as inducible *Smn* alleles by excision of the floxed *pgk-neo* cassette.

### Induction of Smn expression *in vitro*

**Figure 4:**
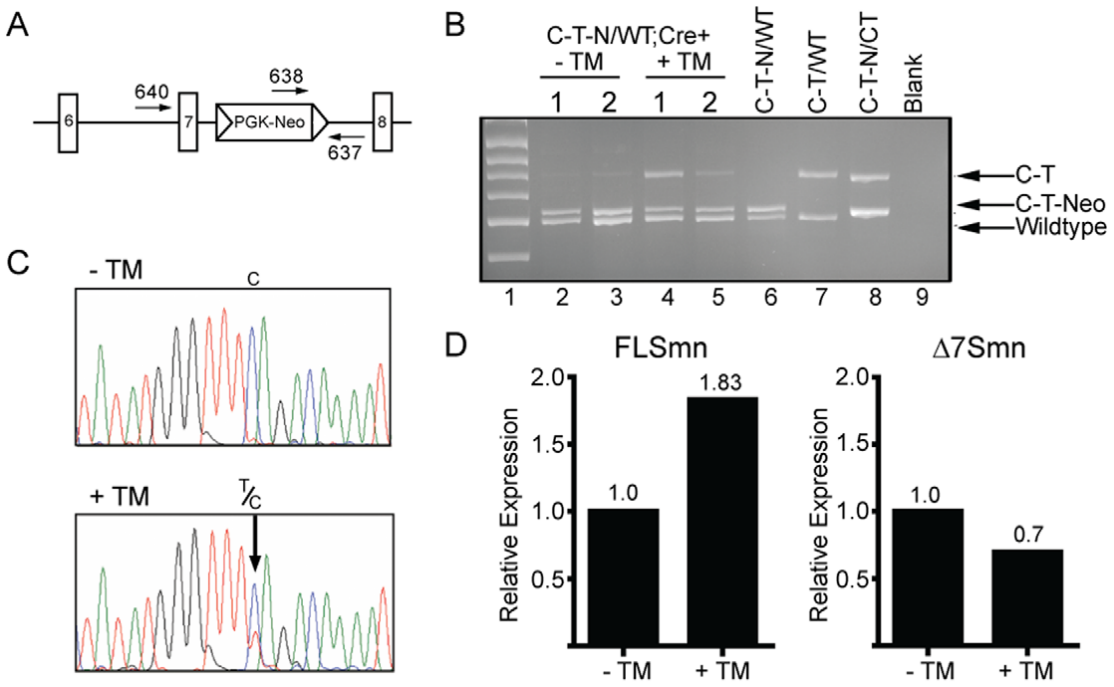
Smn expression is efficiently induced from the *Smn^C-T-Neo^* allele *in vitro*. Two independent primary MEF cells lines were derived from double transgenic embryos (*Smn^C-T-Neo/WT^;Cre^Esr1^*). (**A**) Schematic of the *Smn^C-T-Neo^* allele from exon 6 to exon 8. Arrows represent forward and reverse primers used in the 3-plex PCR reaction to identify *Smn^WT^* (640 & 637), *Smn^C-T-Neo^* (638 & 637), and *Smn^C-T^* (640 & 637) alleles. Primers 640 and 637 do not amplify a product in the presence of *pgk-neo* as the amplicon exceeds the time of elongation. (**B**) 3-plex PCR amplification of DNA from MEF lines 1 and 2 treated for 1 hr with 1 mM tamoxifen (+TM). MEF lines 1 and 2 left untreated (-TM) showed a slight amount of background excision (lanes 2 & 3); however, in the presence of tamoxifen (+TM), they readily amplify the *Smn^C-T^* allele (lanes 4&5). Controls in lanes 6, 7, and 8 were E10.5 embryos harvested to show the indicated genotypes from crosses using germline transmitting *Smn^C-T-Neo^* and *Smn^C-T^* alleles. (**C**) RNA from untreated (-TM) and induced (+TM) MEF cells were amplified by RT-PCR and *FL-Smn* transcripts directly sequenced. Induced MEFs (+TM) produced enough *FL-Smn* transcripts from the mutant C-T allele that could be detected by direct sequencing. The arrow points out the C-T mutation in +TM treated cultures. (**D**) qRT-PCR of *FL-Smn* and *Δ7Smn* from uninduced (-TM) and induced (+TM) cultures. Abbreviations: (TM) tamoxifen (C-T-N/WT;*Cre*+) *Smn^C-T-Neo/+^;Cre^Esr1^* (C-T-N/WT) *Smn^C-T-Neo/+^* (C-T/WT) *Smn^C-T/+^* (C-T-N/C-T) *Smn^C-T-Neo/C-T^*.

**Figure 5:**
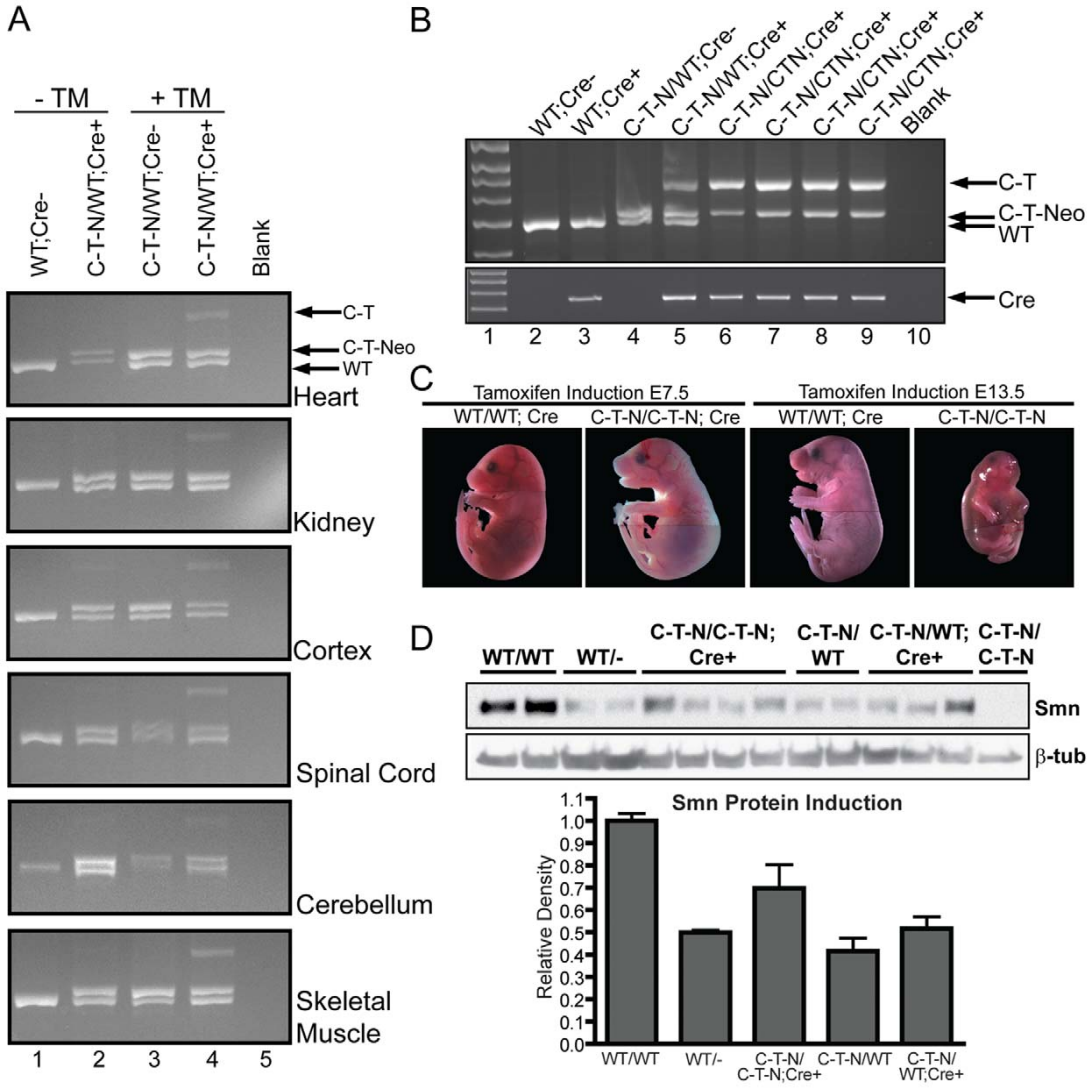
Smn induction in adults and embryos from a single injection of tamoxifen. (**A**) DNA analysis of adult mice i.p. injected with vehicle (corn oil) or TM (9 mg/40 g body weight) using the same 3-plex PCR reaction as shown in Figures 4A and B. Wild type mice (WT;Cre-) only amplified the wild type allele (lane 1). Doubly transgenic mice (*Smn^C-T-Neo/WT^;Cre^Esr1^*) in the absence of TM (-TM) displayed a low basal level of *pgk-neo* excision as has been previously reported for this *Cre* line [37]. In the absence of the *Cre^Esr1^* transgene, *Smn^C-T-Neo/WT^* mice injected with TM could not excise *pgk-neo* (lane 3), in all tissues analyzed, *pgk-neo* excision was only possible and efficient in the presence of *Cre^Esr1^* and TM (lane 4). (**B**) PCR analysis of E18.5 embryos that received a single i.p. dose of TM (3 mg/40 g body weight) to the pregnant dam at E7.5 or E13.5 DNA was genotyped as above to differentiate *Smn^WT^*, *Smn^C-T-Neo^* and *Smn^C-T^* alleles. Arrows identify the appropriate amplicons. (**C**) Photomicrograph of E18.5 embryos induced with TM at E7.5 or E13.5. Lines in photograph show where images were tiled together in Photoshop. (**D**) Western blot and semi-quantitative densitometry of protein extracted from brain tissue of induced and control E18.5 embryos. A small amount of protein was able to be extracted from severely deformed *Smn^C-T-Neo/C-T-Neo^* embryos identified as “escapers” for comparison to induced *Smn^C-T-Neo/C-T-Neo^;Cre^Esr1^* rescued embryos. Semi-quantitative densitometry was performed on a separate blot using the same samples shown and normalized to β-tubulin, without the uninduced mutant. Protein levels from induced homozygous embryos, *Smn^C-T-Neo/C-T-Neo^;Cre^ESR1^*, (0.7±0.10) was greater than *Smn^WT/-^* (0.5±0.2). Abbreviations: (WT) *Smn* wild type allele, (*Cre+* and *Cre-*) presence or absence of *Cre^Esr1^*, (C-T-N/WT) *Smn^C-T-Neo/WT^*, (C-T-N/C-T-N) *Smn^C-T-Neo/C-T-Neo^*, (C-T) *Smn^C-T^* allele, (C-T-Neo) *Smn^C-T-Neo^* allele, (TM) tamoxifen.

We determined the ability of our progenitor alleles to induce Smn levels through excision of the floxed *pgk-neo* cassette *in vitro* using primary murine embryonic fibroblasts (MEFs). For these experiments, we used our *Smn^C-T-Neo^* allele in combination with a tamoxifen (TM) inducible *Cre* allele, *Cre^Esr1^* [37]. We established and cultured MEF lines from two *Smn^C-T-Neo^* ^/WT^;*Cre^Esr1^* embryos and compared untreated MEF cultures to those treated with TM. Excision of floxed *pgk-neo* by *Cre^Esr1^* was monitored at the DNA level by three primer PCR analysis (Figure 4A). The reaction amplified all three possible *Smn* alleles: *WT* (470 bp), *C-T-Neo* (500 bp) and *C-T* (577 bp) (Figure 5B). MEFs treated with TM demonstrated excision of the floxed *pgk-neo* cassette from the *Smn^C-T-Neo^* allele (Figure 4B, lanes 4 and 5). A low level of excision was also noted in cells without TM treatment; we attributed this to the previously reported <0.1% spontaneous C*re* activity that was reported for this line [37] (Figure 4B, lanes 2 and 3). We then correlated *pgk-neo* excision with an increase in *FL-Smn* expression by directly sequencing RT-PCR products from treated and untreated MEFs. A clearly discernable “T” nucleotide was present in those cells treated with TM and absent in the untreated cultures indicating an increase of Smn expression specifically from our mutant allele (Figure 4C). This was substantiated and quantified by qRT-PCR analysis which showed an 83% increase in *FL-Smn* transcripts and a 30% reduction in *Δ7Smn* transcripts between treated and untreated cultures (Figure 4D). Therefore, addition of tamoxifen to cultures of *Smn^C-T-Neo^* ^/WT^;*Cre^Esr1^* MEFs increased *FL-Smn* expression through excision of the floxed *pgk-neo* cassette in *Smn* intron 7 and demonstrates the inducible nature of these alleles *in vitro*.

The ability of our *Smn* alleles to be *Cre* responsive in post natal somatic tissues was determined by single intraperitoneal (i.p.) injections [9 mg/40 g body weight] of TM or vehicle (corn oil) to 2–4 month old *Smn^C-T-Neo^* ^/WT^;*Cre^Esr1^* mice. They were euthanized five days post-injection and DNA from kidney, spinal cord, skeletal muscle, forebrain and cerebellum were used as template in PCR to determine floxed *pgk-neo* excision. Vehicle injected *Smn^C-T-Neo/WT^;Cre^Esr1^* mice had a low level of DNA excision consistent with background levels of *Cre^Esr1^* (Figure 5A, lane 2). *Smn^C-T-Neo^* ^/WT^ mice without *Cre^Esr1^* showed no excision of the floxed *pgk-neo* cassette (Figure 5A, lane 3). In contrast, excision occurred in all tissues examined from *Smn^C-T-Neo/WT^;Cre^Esr1^* mice after a single i.p. injection (Figure 5A, lane 4), hence somatically changing the *Smn^C-T-Neo^* allele to a *Smn^C-T^* allele.

### Induction of Smn in *Smn^C-T-Neo^* ^/*C-T-Neo*^;*Cre^Esr1^* embryos early, but not late, rescues embryonic lethality

The early lethality of *Smn^C-T-Neo/C-T-Neo^* embryos was determined to be caused by low Smn levels, thus we next sought to determine when during development, if at all, we could increase Smn levels and rescue the embryonic lethality phenotype. To this end we crossed *Smn^C-T-Neo/WT^* females to *Smn^C-T-Neo/WT^;Cre^Esr1^* males in order to produce *Smn^C-T-Neo/C-T-Neo^*;*Cre^Esr1^* embryos. Without TM treatment these embryos should undergo complete resorption between E12.5 and E15.5 (Figure 2 and Table 1). Pregnant females were injected with TM [3 mg/40 g body weight] at either E7.5 or E13.5. Only two litters (9 pups total) survived birthing from 7 dams injected at E7.5 and visibly pregnant at late gestation. Of these 9 pups, 1 was a *Smn^C-T-Neo/C-T-Neo^*;*Cre^Esr1^* pup and of similar size and appearance to its littermates. However, in both litters the dams failed to lactate and fostering was unsuccessful. Birthing and lactation problems are a known common side effect of TM administration, especially when given early during pregnancy [37]. To avert this problem we harvested embryos at E18.5, a late point in gestation, and well after a stage in development (E15.5) in which no viable homozygous *Smn^C-T-Neo/C-T-Neo^* embryos had previously been identified (Table 1). DNA prepared from yolk sacs or tails was used for PCR-based genotyping with the same 3-plex PCR assay used in Figures 4 and 5A. This allowed us to identify and determine the efficiency of the floxed *pgk-neo* cassette excision. The presence or absence of the *Cre^Esr1^* transgene was determined in a separate reaction. In all embryos that carried both the *Smn^C-T-Neo^* allele and *Cre^Esr1^* transgene, the injection of TM induced excision of floxed *pgk-neo*, thus somatically changing the *Smn^C-T-Neo^* allele to a *Smn^C-T^* allele (Figure 5B, lanes 5-9). Smn induction of 4 litters at E7.5 resulted in a total of 32 embryos at E18.5 and five were found to be *Smn^C-T-Neo/C-T-Neo^*;*Cre^Esr1^* embryos (Table 3). All five of these embryos were viable, of similar size and indistinguishable from control embryos (Figure 5C). In contrast, TM injection of 6 litters at E13.5 resulted in no viable *Smn^C-T-Neo/C-T-Neo^*;*Cre^Esr1^* embryos at E18.5 (0/42) (Table 3). However, in each of these crosses we did identify a *Smn^C-T-Neo/C-T-Neo^* embryo that was negative for the *Cre^Esr1^* transgene although both were severely deformed, nonviable and undergoing resorption (Figure 5C). Chi-square statistics were used to compare the genetic ratios of induced embryos to expected normal mendelian ratios with the assumption that *Smn^C-T-Neo/C-T-Neo^* embryos would not be present at E18.5. Genotype ratios from embryos treated at E13.5 were not statistically significant from expected ratios, p = 0.84. However, the genotype ratios of embryos treated at E7.5 were significant, p = 0.05 (Table 3). Thus, increasing Smn levels rescues the embryonic lethality phenotype of *Smn^C-T-Neo/C-T-Neo^*;*Cre^Esr1^* embryos when induced early, but not late in development.

**Table 3:** Genotypes of *Smn* embryos at E18.5 exposed to tamoxifen at E7.5 or E13.5.

	# of litters	Total # pups	WT (%)	WT;Cre (%)	C-T-N/WT (%)	C-T-N/WT; Cre (%)	C-T-N/C-T-N (%)	C-T-N/C-T-N; Cre (%)	Unknown
E7.5 injection, E18.5 harvest	4	32	3 (9)	5 (16)	3 (9)	11 (34)	1 (3)	5 (16)	4 (13)
E13.5 injection, E18.5 harvest	6	42	6 (14)	6 (14)	10 (24)	10 (24)	1 (2)	0 (0)	9 (21)

(χ^2^, E7.5 injection p = 0.051; E13.5 injection p = 0.84).

To quantify the level of Smn in these *Smn^C-T-Neo/C-T-Neo^*;*Cre^Esr1^* embryos, we performed western blot analysis and compared them to a series of control, age-matched embryos that titred Smn levels between 100% and 50%. In addition, we were able to extract a small amount of protein from one of the non-viable *Smn^C-T-Neo/C-T-Neo^* embryos for comparison to our induced embryos (Figure 5D). However, without *Cre* mediated excision of the floxed *pgk-neo* cassette, Smn levels in this embryo were below the limit of detection even though the loading control, β–tubulin was visualized (Figure 5D). Densitometry was performed on a separate western blot using the same samples for the other genotypes (Figure 5C). The excision of the floxed *pgk-neo* cassette in *Smn^C-T-Neo/C-T-Neo^*;*Cre^Esr1^* +TM embryos increased Smn protein levels to 70% of wild type expression (Figure 5D). This is greater than the 50% of Smn protein detected in heterozygous *Smn* null (*Smn^WT/^* ^-^) embryos which develop normally and have a normal postnatal lifespan (Figure 5D) [1]. Furthermore, this is greater than the level of Smn expressed in our *Smn^C-T-Neo^* and *Smn^2B-Neo^* progenitor lines of mice that have a normal lifespan and are without phenotype.

## Discussion

We specifically designed the targeting vectors used in this study to have a triple function so that a series of *Smn* alleles could be generated. The first was the introduction of mutations within known *Smn* exon 7 ESEs to mimic *SMN2* exon 7 splicing and attenuate *Smn* expression. The second was the placement of the positive selection cassette within intron 7 with the aim of further diminishing the amount of Smn generated from these targeted alleles. It has previously been shown that selection cassettes located within introns or regulatory regions, such as *pgk-neo* that we used here, can hinder expression of targeted genes via transcriptional and/or translation interference either by design ([38]; additional examples cited in [39]) or inadvertently [40], [41], [42], which sometimes proves to be serendipitous [42]. The level of interference can be varied depending upon the orientation of the selection cassette if it has cryptic splice donor and acceptor sites, like the *neo* gene, [39] or they can be engineered. Here we used *pgk-neo* in the inverted orientation to *Smn* transcription as the cryptic splice sites were stronger. The third and final function of our design was to flank *pgk-neo* with *lox*P sites so that it could be removed with Cre recombinase. This allows the specific analysis of our introduced mutations within the context of a minimally altered genomic locus. Since the *neo* cassette hindered Smn expression as we designed, our alleles can be used to induce Smn expression while still mimicking *SMN2* exon 7 alternative splicing. The conceptual properties of this targeting design are applicable to almost any gene targeting strategy and warrant consideration for those embarking on new projects.

Our allelic targeting strategy provides the opportunity to generate three mouse lines from each single allele. The first is the original progenitor alleles, S*mn^C-T-Neo^* and *Smn^2B-Neo^*. The second is these alleles in combination with a tissue specific or inducible *Cre* transgene for future time and cell-specific induction experiments. Finally, mouse lines possessing the point mutations themselves, *Smn^C-T^* and *Smn^2B^*, can be produced. Here we have focused on characterizing the first two lines of mice.

Mice heterozygous for the S*mn^C-T-Neo^* and *Smn^2B-Neo^* alleles were normal. When homozygous, these alleles caused embryonic lethality, even though full-length transcripts and small amounts of Smn protein could be detected. This result illustrates once again that a minimum amount of Smn is required by all cell types [1], [34], [35], [43], [44], and this appears to be about 5% during development in mice. Although we found no significant difference in mendelian ratios of homozygous mutants for either allele at E9.5, morphologically it was clear that the embryos were dead or extremely growth retarded and their ability to develop corresponded to their Smn levels. Embryonic lethality during this period of development is commonly caused by potential failures in gastrulation, defects in extra embryonic membrane functions such as vasculogenesis or hematopoiesis and/or cardiovascular failure [45]. Considering these possibilities, we were able to visualize swollen pericardial sacs in several of our S*mn^C-T-Neo/C-TNeo^* homozygous mutants at E11.5 (data not shown) or E12.5 that were not grossly deformed.

Functionally and molecularly the homozygous S*mn^C-T-Neo/C-T-Neo^* and *Smn^2B-Neo/2B-Neo^* hypomorphs are less severe than *Smn^Δ7/Δ7^* homozygotes as they express small amounts of fully functional Smn protein and live slightly longer (E12 and E9 vs E7) [34], [35]. The S*mn^C-T-Neo/C-T-Neo^* homozygotes are generally less severe than the *Smn^2B-Neo/2B-Neo^* homozygotes due to the nature of the introduced mutations within exon 7 since the C-T mutation produces more *FL-Smn* transcripts than the 2B mutation. During the course of our molecular analyses there were two unexpected observations. First, we found that *Δ7Smn* transcripts from *Smn^2B-Neo/2B-Neo^* embryos were extremely high (12.44±3.44) vs. heterozygous *Smn^2B-Neo^* embryos (1.72±0.23). This may seem odd at first glance, but SMN together with other proteins functions in the assembly of small nuclear ribonucleoproteins (snRNPs), which are critical for pre-mRNA splicing [46], [47]. It has previously been demonstrated that low levels of SMN cause changes in snRNP abundance and splicing alterations [48], [49], [50], so it is possible that SMN regulates its own splicing of *SMN2* exon 7. In fact, Jodelka et al. (2010) [51] has recently identified a feedback loop in which low SMN levels exacerbate *SMN2* exon 7 skipping. This result helps explain the high degree of *Δ7Smn* transcripts from our *Smn^2B-Neo/2B-Neo^* embryos since they produce only 1-3% Smn protein. The second observation was the low amount of Smn protein (<5%) as compared to *FL-Smn* transcripts (10-20%) in our homozygous embryos. We predict this is caused by a combination of the degenerative state of the embryos, lack of higher order Smn complexes that can stabilize the protein, and the presence of the floxed *pgk-neo* cassette (as we designed), which creates hybrid *Smn/neo* transcripts and exerts transcriptional and translational repression. We show that removal of the *pgk-neo* cassette is able to relieve this repression.

As progress in developing SMN-dependent therapies moves forward, our understanding of when and where SMN is required for severe, intermediate and mild forms of SMA becomes important. This can be addressed using animal models since they can be genetically manipulated. SMA has already been modeled in a number of different organisms and each has its strengths [52], [53], but the most widely used to date have been SMA mouse models. The mouse models fall into two categories:1) transgenics bred onto *Smn* null or Δ7 backgrounds to rescue the embryonic lethality of homozygous *Smn* mutants to produce varying degrees of SMA severity, and 2) modifications of the endogenous *Smn* locus that reduce Smn expression. One conditional allele of *Smn* has been generated, a floxed allele of *Smn* exon 7, which can be combined with varying *Cre* transgenes for the temporal or spatial depletion of Smn. Although this is a very useful allele and conditional depletion experiments have been done [34], [43], [44], SMA is due to a paucity of Smn protein not complete absence of Smn protein. Currently lacking, and which we begin to fulfill here, is a panel of different *Smn* inducible alleles that can be used in future experiments to address the cellular and temporal requirements of Smn induction in varying SMA disease severities.

To validate the potential of S*mn^C-T-Neo^* and *Smn^2B-Neo^* to be utilized as inducible alleles, we used S*mn^C-T-Neo^* as a proof of concept allele. Using S*mn ^C-T-Neo^* in combination with a tamoxifen inducible *Cre* transgenic mouse, *Cre^Esr1^*, we derived primary MEF lines and proved that only in the presence of tamoxifen was Smn induced. This illustrates the efficacy of Smn induction *in vitro* and the potential of using these lines to establish *Smn* inducible culture models. In addition, a single i.p. injection of tamoxifen was able to excise the floxed *pgk-neo* cassette in somatic tissues of adult mice and embryos. Hence, the alleles can be used in future somatic experiments to assist in determining the cellular requirements of Smn. This can be achieved through genetic crosses of tissue-specific *Cre* lines or as shown here, through injections if the *Cre* lines are tamoxifen inducible. In addition, if the S*mn ^C-T-Neo^* and/or *Smn^2B-Neo^* is used as the *Smn* mutant background in combination with *SMN2* transgenic mice to achieve postnatal survival, new SMA models can potentially be generated with varying degrees of severity. Thus, the timing requirements of Smn inductive therapies could be addressed postnatally in severe, intermediate and mild forms of SMA. We are currently working to develop these models and determine this.

In a final experiment, we addressed whether the embryonic lethality of S*mn^C-T-Neo/C-T-Neo^* embryos could be rescued. We found that we could, if induction occurred early, around gastrulation (E7.5), but not late (E13.5), and the mutant embryos that were induced at E7.5 expressed ∼70% Smn protein. This level of Smn protein is well above that of *Smn^+/−^* mice that have a normal lifespan [1] or heterozygous SMA model mice [*(SMN2)Ahmb89^+/+^;Smn^+/−^*; Jax strain 5024], which have no motor neuron loss and a normal lifespan [54]. At this point our work is unable to address the dosage and timing of Smn expression necessary to alleviate postnatal SMA disease, which is the time period when all, but the very most severely affected, SMA patients present with symptoms. However our results are important as they are consistent with the few pharmacological or gene therapy studies where a therapeutic window of opportunity to demonstrate a survival or functional benefit exists in severe SMA [15], [17], [29]. We all show that the earlier the intervention, the better the outcome. The mounting results of these types of studies are important points of consideration as clinical trials based on SMN-inductive therapies are designed, especially for the most severe SMA patients. It will be important for future studies to add and extend this work. For example, a paramount point is whether SMN inductive therapies can provide benefit during the lag/plateau phase of disease or prevent further deterioration of function from the point of treatment. We believe that the alleles we have generated here will be useful in developing the appropriate models to answer this question.

In conclusion, the gene targeting strategy that we utilized is applicable to almost all gene targeting projects. The strategy provides the potential to generate multiple lines of mice, including an inducible allele, from a single targeting event for the cost of extra effort put into the planning stage of vector design. As proof, we generated an allelic series of *Smn* mice. They produce small amounts of Smn, mimic *SMN2* splicing and are inducible. While these are the first inducible *Smn* alleles to be described in the literature, other groups are also working to generate inducible *Smn* mice using different strategies such as Tet-on/off systems or other *Cre*-inducible alleles. As proven with the various transgenic models of SMA, all will play important and complimentary roles as we move forward to understand Smn function in health and disease, and progress towards developing a therapy for SMA.

## Materials and Methods

### Ethics Statement

All studies performed on mice were in accordance with the Institutional Animal Care and Use Committee regulations in place at Children's Memorial Research Center and specifically approved under protocols 2007-15, 2006-22 and 2008-03.

### Construction of targeting vectors and generation of Chimeras

The replacement vectors, p(Smn^C-T-Neo^) and p(Smn^2B-Neo^) are shown schematically in Figure 1A. The total length of homology between the targeting vector and the endogenous *Smn* locus is 6.5 kb; the long arm is 5.0 kb and the short arm is 1.5 kb. The positive selection cassette, *flox-pgk-neo* was inserted into the unique BamHI site 180 bp distal to exon 7. This was done to increase the chance of homologous recombinant clones containing the C-T transition in exon 7 or the 2B mutation (GGA-TTT) in exon 7, since recombination is unlikely to occur within such a short distance between our modification and the positive selection cassette. The mutations were introduced via site directed mutagenesis of a smaller fragment and then re-subcloned into the targeting construct to avoid potential mutations generated by PCR. In total, after Cre excision of the *pgk-neo* cassette, ∼90 bp will remain, of which, 34 bases correspond to the remaining loxP site. The 90 bp of sequence is located sufficiently distal to exon 7 and should not affect regulation or processing of the *Smn* ^C-T-Neo^ and *Smn* ^2B-Neo^ transcripts. The targeting constructs were electroporated into 129 ES cells and 4 independent homologous recombinant clones for each construct were identified. Two from each construct were used to generate chimeras by microinjecting ES cells into C57Bl/6 blastocysts.

### Mice and Animal Care

Animals were kept in a controlled vivarium at 25°C and 50% humidity in a 12 hour light/12 hour dark photoperiod and monitored for health. Colonies were maintained by breeding mice heterozygous for the *Smn* mutant alleles. The official name for *Smn^C-T-Neo/+^* mice is *Smn^tm2Cdid^*. It is being placed at The Jackson laboratory in both C57BL/6 and FVB/N genetic backgrounds but currently lacks official strain designation. The official name of the *Smn^2B-Neo/+^* mice is *Smn^tm1Cdid^* and it is also being placed at The Jackson Laboratory in both C57BL/6 (Jax strain 008838) and FVB/N (JAX 008837) genetic backgrounds. The lines will be made available once they are on congenic C57Bl/6 and FVB/N genetic backgrounds.

### Genotyping reactions

All PCR and RT-PCR experiments were performed on an Eppendorf Mastercycler®. *Smn^C-T-Neo/+^* and *Smn^2B-Neo/+^* adults (Table 1) were genotyped using forward primer #638 (5′-AATGTG TGC GAGGCCAGAGG-3′) found within the PGK promoter and reverse primer #637 (5′-TTTGGCAGACTT TAGCAGGGC-3′) within intron 7 multiplexed with an internal control set of primers that amplified *Smn* exon 4 using primers #379 (5′- AGGCGT TGAATGACATTCTC) and #380 (5′-GCCATACAAAGTGTTCACAC). Reaction conditions: 94°C/45 sec, 60°C/45 sec, 72°C/45 sec, 35 cycles. C-T-Neo or 2B-Neo alleles produced a 519 bp amplicon and the internal control amplified a 697 bp product. PCR products were resolved on a 1.5% agarose gel.

*Smn^C-T-Neo^* and *Smn^2B-Neo^* allelic embryos (Table 1) were genotyped using a three primer PCR reaction. The primers include *Smn* intron 6 forward, #721 (5′-TATCACTAAGTTGGGCGA AAG GG-3′), *Smn* intron 7 reverse, #636 (5′-TTTGGCAGACTT TAGCAGGGC-3′), and PGK promoter reverse primer #638 (5′-AATGTGTGCGAGGCCAGAGG-3′). Reaction conditions: 94°C/45 sec, 64°C/45 sec, 72°C/1 min, 35 cycles. The product sizes for the C-T-Neo and 2B-Neo alleles are 519 bp and 700 bp for the WT allele.

Analysis of *pgk-neo* excision was performed on MEF colonies, induced adults and embryos using a 3- primer PCR reaction with primers #638, intron 6 forward primer # 640, (5′- AACTCCGGGTCCTCCTTCCT) and #637 to amplify *Smn^WT^* (470 bp), *Smn^C-T-Neo^* (500 bp), and *Smn^CT^* (577 bp) alleles. *Cre* amplification utilized forward primer #162 (5′-CGCCGCATAACC AGTGAAAC-3′) and reverse primer #163 (5′-ATGTCCAATTTACTGACCG-3′) to amplify a 335 bp fragment.

*Smn* exon 7 was amplified for direct sequencing using primers forward intron 6 primer #58 (5′-CCTCCTTCCTCCTCATCTCAG-3′) and reverse intron 7 primer #62 (5′- AATTATACAAAAGGTAAAATTAGC) under standard cycling conditions. PCR products were purified with Montage PCR clean up column (Millipore) and sequenced using intron 6 primer #59 (5′-TCCAGCCGGGCTTGAATT).

### RNA Transcript Analysis

RNA was extracted from tissues using TRIzol reagent (Invitrogen) in accordance with the manufacturer's directions. Samples were then treated with TURBO DNA-free reagents (Ambion) and first strand cDNA was prepared using SuperScript™ II Reverse Transcriptase (Invitrogen) and random hexamer primers (Roche, Nutley, NJ) according to the manufacturer.

The RT-PCR reaction to amplify both full length *Smn* and Δ7*Smn* mRNA used primers 455 and 495. All reactions used 100 ng cDNA in a mix of 10X buffer (20 mM Tris-HCl (pH 8.4), 50 mM KCl), 200 µM dNTP mix, 3 mM MgCl_2_, 7.5pM primers, and 1U taq polymerase. RT-PCR conditions were as follows: 94°C/45 sec, 60°C/45 sec, 72°C/45 sec, 30 cycles. RT-PCR reaction used to identify the full length products of homozygous mutant embryos (Fig. 3*A*) was performed using an exon 5 forward primer #741 (5′- TCCCTTCAGGACCACCAATA) and an exon 8 reverse primer #495. Reaction conditions were: 94°C/45 sec, 60°C/45 sec, 72°C/45 sec, 35 cycles. *FL-Smn* transcripts from MEFs that were used as a template in sequencing were amplified using primers #741 and# 495. Δ7*Smn* transcripts were amplified using exon 5-6 forward primer #490 (5′-CTTCAGGACCACCAATAA TC) and exon 6-8 reverse primer, #500 (5′-GACAGAGCTGAACAATAT). Both were sequenced with primer #490.

qRT-PCR was performed on cDNA prepared as described above. All reactions were performed in triplicate in a 20 ul final reaction volume. Since the assay on demand primer and probe available from ABI for *Smn* exon 7 are too close or overlap the C-T and 2B mutations and could hinder interpretation of results, we designed and validated *FL-Smn* and Δ*7Smn* assays to specifically amplify *Smn* transcripts with similar efficiencies between the WT, C-T and 2B mutations and not cross react with human SMN. Once validated these assays were prepared by ABI in a 20X mastermix to be similar to an assay on demand. *FL-Smn* was amplified and detected with primer #ABI-1, Smn678F (5′-TGGCTACCACACTGGCTACTATATG), primer #ABI-2, Smn678R (5′- GACACCCCATCTCCTGAGACA) and probe ABI-3, Smn678M1 (6FAM-CATACAAATTAAGAAGTTCAGC). Δ*7Smn* transcripts were amplified and detected with primer #ABI-4, Smn_EX6_8F (5′- GGCAGTATGCTAATCTCTTGGTACA), primer #ABI-5, Smn_EX6_8R (5′- CGACACCCCATCTCCTGAGA) and probe #ABI-6, Smn_EX6_8M1 (5′- 6FAM-CAGAGCTGAACCATATAGTAGC). mGapdh On Demand Assay (Applied Biosystems) was used as an internal control for normalizing data. All qRT-PCR reactions were run using an Applied Biosytems 7500 Fast Real-Time PCR System utilizing the following conditions: 1 cycle: 2 min 50°C, 1 cycle: 10 min 95°C, 40 cycles: 15 sec 95°C, 1 min 60°C. All data was analyzed using the ΔΔCt method.

### Protein isolation, western blot and densitometry

Tissues and embryos were homogenized as previously described [55]. Primary antibodies used were mouse monoclonal antibodies to Smn (BD Translabs) at 1∶5,000 and β-tubulin at 1∶20,000. Polyclonal rabbit actin at 1∶1,000. The secondary antibody, goat anti-mouse (BioRad) or goat anti-rabbi (BioRad) were used at 1∶10,000. Membranes were exposed to chemiluminescence (ECL Western Blotting Detection Reagents, Amersham Biosciences) and developed on Kodak film. Blots were imported into Adobe Photoshop using Microtek 1000XL scanner and quantified through densitometry by Openlab 5.0 software. The Li-Cor Odyssey System was used to quantify embryo extracts in Figure 3 according to the instructions of the manufacturer (Li-Cor Biosciences). Briefly, proteins were resolved on a 4–12% Bis Tris NuPage pre-cast gel system using MES running buffer and transferred to nitrocellulose using the I-Blot transfer system (Invitrogen). Bound antibodies were detected using IRDye800CW-conjugated goat anti-mouse IgG (Li-Cor). The intensity of each band was measured and normalized to that obtained from Tubulin.

### Whole Mount Embryo Images

Pregnant female mice were sacrificed by CO_2_ asphyxiation followed with cervical dislocation. Embryos at either E9.5, E12.5 or E18.5 were captured on a Leica upright dissecting microscope and digital camera at 2.0X, 1.0X, and 0.8X magnifications respectively. Embryos at E11.5 were captured at 1.0X. Photographs were taken at a 300dpi, 8 bit/channel quality.

### *pgk-neo* excision by TM in MEFs

Pregnant females were sacrificed with CO_2_ asphyxiation at 14.5 days post coitum (dpc). Embryos were individually eviscerated and rinsed in 1X PBS for MEF preparation. Yolk sac was collected for DNA to identify *Smn^C-T-Neo/+^;Cre^Esr1^* embryos. Embryos were finely minced with a straight edge razor and incubated in trypsin at 37°C. Debris were removed by resuspending cells in 15cc tube containing cell growing media (Earle's alpha-MEM, 10% FBS, 1% pen/strep, 1% non-essential amino acids, 1% L-glutamine) and allowing large debris to collect at the bottom. The supernatant was plated and cells were passaged 4 times before those with the genotype *Smn^C-T-Neo/+^;Cre^Esr1^* were used for experiments. TM (1 mM) was added to cultures for 1 hr to induce CRE activity. Control cells received no TM. After 24 hours cells were harvested for DNA and RNA as previously described for tissue samples.

### *pgk-neo* excision by TM in adult and embryonic mice

Injection of TM to both adult and embryo used here, were carried out as previously described [37]. TM was diluted to 10 mg/ml in corn oil. Adults were injected with 9 mg TM/40 g body weight and sacrificed five days later. Tissues harvested included spinal cord, skeletal muscle (gastrocnemius and quadriceps), cortex, cerebellum, heart and kidney. Both DNA and RNA were extracted as previously described. For embryonic induction, pregnant females were injected at 7.5 dpc or 13.5 dpc with 3 mg TM/40 g of body weight. At 18.5 dpc, females were sacrificed with CO_2_ asphyxiation and embryos dissected individually. From each embryo the lung was harvested for DNA and the remainder flash frozen in liquid nitrogen and stored at −80°C.
